# Clinical outcomes of salvage treatment in lymph node-positive prostate cancer patients after radical prostatectomy

**DOI:** 10.1371/journal.pone.0256778

**Published:** 2021-09-10

**Authors:** Dowook Kim, Dong-Yun Kim, Jae-Sung Kim, Sung Kyu Hong, Seok-Soo Byun, Sang Eun Lee

**Affiliations:** 1 Department of Radiation Oncology, Seoul National University Hospital, Seoul National University College of Medicine, Seoul, Republic of Korea; 2 Department of Radiation Oncology, Seoul National University Bundang Hospital, Seoul National University College of Medicine, Seongnam, Republic of Korea; 3 Department of Urology, Seoul National University Bundang Hospital, Seoul National University College of Medicine, Seongnam, Republic of Korea; Tata Memorial Centre, INDIA

## Abstract

**Introduction:**

The optimal salvage treatment strategies for lymph node-positive (LNP) patients after radical surgery have not been clearly defined in prostate cancer with biochemical recurrence or persistence of elevated prostate-specific antigen (PSA). In this study, we compared the clinical outcomes of two different salvage treatments, androgen deprivation therapy (ADT) alone versus ADT with radiotherapy (RT). We also investigated prognostic factors that could support the use of ADT with RT in LNP prostate cancer.

**Materials and methods:**

We retrospectively reviewed 94 LNP prostate cancer patients who underwent radical prostatectomy (RP) followed by salvage treatment between 2004 and 2018. Salvage treatments involved either ADT alone or ADT with RT according to the clinical judgment of the physician. We analyzed clinicopathological and treatment factors related to 2^nd^ biochemical failure (2^nd^ BCF), clinical progression (CP), and progression-free survival (PFS). The cumulative failure after salvage treatment was defined as including both 2^nd^ BCF and CP.

**Results:**

The median duration of follow-up was 55 months (interquartile range, 35–97 months). Thirty-seven (39.4%) patients were treated with ADT alone, and 57 patients (60.6%) were treated with a combination of ADT with RT. During follow-up period, the incidence of failure after salvage treatment in the ADT alone group and the combined treatment group was 89.2% and 45.6%, respectively (HR, 22.4; 95% CI 5.43–92.1; P < 0.001). The combination of ADT with RT was associated with better 2^nd^ BCF and PFS than ADT alone (*P* = 0.007 and *P* = 0.015, respectively). In multivariate analyses, number of positive LN ≥ 2 and PSA nadir ≥ 0.005 ng/ml after RP were associated with poor 2^nd^ BCF, CP, and PFS after salvage treatment. Salvage by combined ADT plus RT showed better 2^nd^ BCF and PFS than ADT alone. Specifically, patients with number of positive LN ≥ 2 or PSA nadir ≥ 0.005 ng/ml after RP showed better 2^nd^ BCF (*P* = 0.004) or PFS (*P* = 0.011) when treated with ADT plus RT rather than ADT alone.

**Conclusions:**

In patients with LNP prostate cancer, salvage ADT plus RT improved 2^nd^ BCF and PFS compared to ADT alone. In particular, when the patients had more than two positive lymph nodes or PSA nadir ≥ 0.005 ng/ml after RP, ADT with RT seems to be a more beneficial salvage treatment resulting in better 2^nd^ BCF and PFS.

## Introduction

Radical prostatectomy (RP) is one of the first-line treatment option for locally advanced prostate cancer. Of all prostate cancer patients who receive RP and pelvic lymph node dissection (PLND), 3–14% have pathological lymph node-positive (LNP) disease [1–3]. Several previous studies showed that LNP was associated with poor prognosis in prostate cancer patients who received RP as initial treatment [4–6]. Nonetheless, the optimal management of patients with LNP still remains unclear. The National Comprehensive Cancer Network (NCCN) guidelines described several options of adjuvant therapy for LNP patients: androgen deprivation therapy (ADT), observation or addition of pelvic external beam radiation therapy (RT) to ADT [7]. Giving immediate ADT was recommended as an adjuvant treatment compared with deferred ADT in LNP disease [8], and observation can be chosen depending on the judgment of the physician in consideration of the risk stratification of the tumor itself and the life expectancy of the individual patient. It has also been reported that RT can be used in combination with ADT, but this is based on a lower-level evidence [7]. Meanwhile, after selecting the observation policy in patients with LNP, salvage treatments can be offered when symptomatic progression or elevation of prostate-specific antigen (PSA) is confirmed. In patients of LNP prostate cancer, the rates of biochemical recurrence (BCR) and clinical recurrence at 10 years after RP have been reported as 45% and 30%, respectively [9]. In addition, 26–54% of patients with LNP prostate cancer have persistent elevated PSA even after receiving RP [10, 11]. Such patients showing BCR or persistent PSA need salvage treatments, including ADT alone, RT alone or ADT in combination with RT [12–15]. Song et al. reported that RT ± subsequent ADT represents a significant benefit in clinical progression (CP)-free survival compared to ADT alone in recurrent prostate cancer patients receiving salvage treatment [14]. Recently, a study has shown that early salvage RT may be the best option for patients with BCR after RP [13], but no consensus has been reached yet. In particular, most of these salvage options are aimed at node-negative prostate cancer patients, so the results may not be generalized in patients with LNP prostate cancer. Considering the high risk of recurrence in patients with LNP prostate cancer and the scarcity of relevant studies analyzing LNP prostate cancer patients after RP, further research of salvage treatment may be more important in patients with BCR or persistent elevated PSA.

This study aimed to evaluate the clinical outcomes of salvage treatments between ADT alone and ADT with RT and identify clinicopathological and treatment factors affecting prognosis in LNP prostate cancer.

## Materials and methods

### Patient eligibility

**Fig 1:**
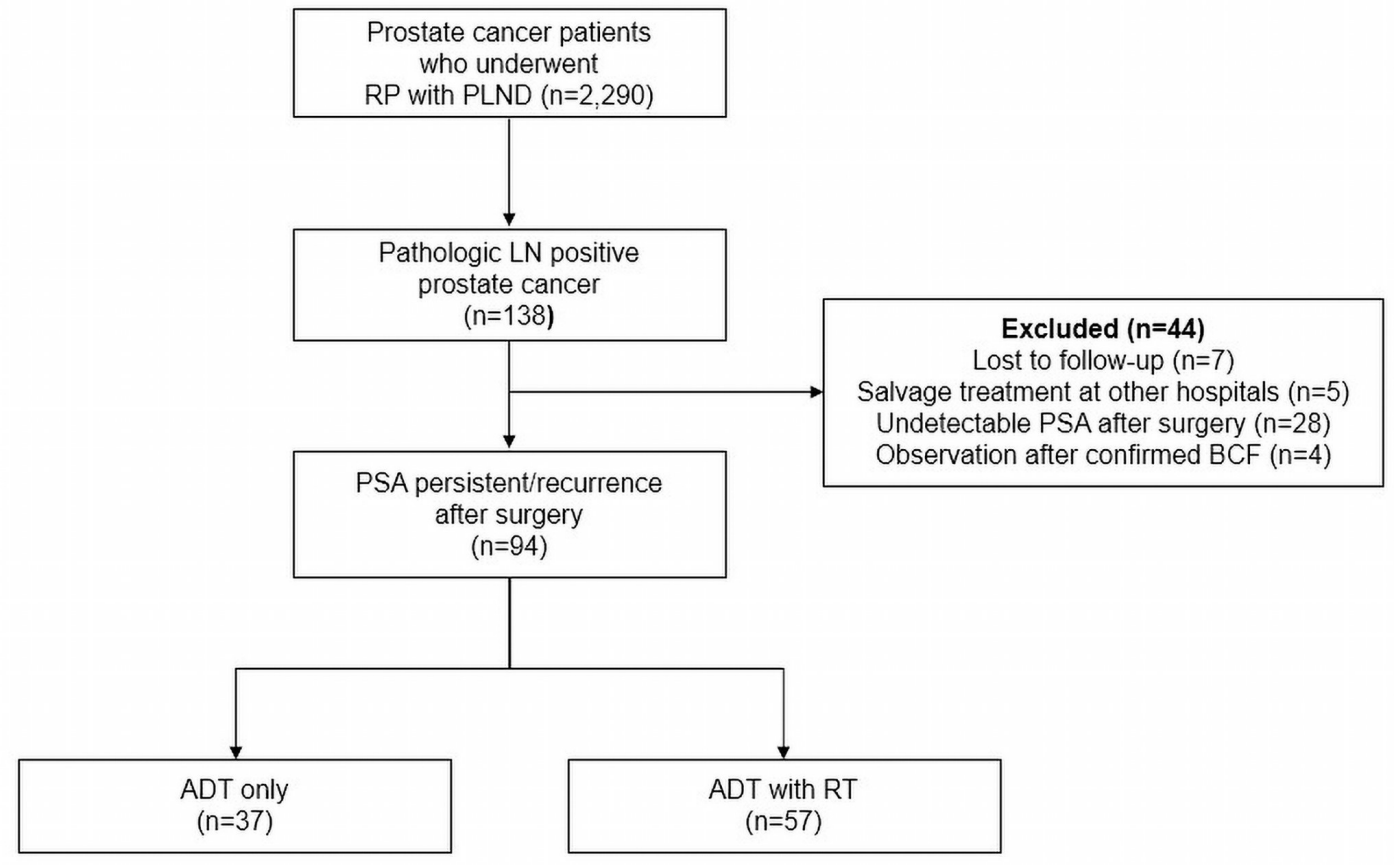
Flowchart of included and excluded patients and group assignments. Abbreviations: ADT, androgen deprivation therapy; LN, lymph node; PLND, pelvic lymph node dissection; PSA, prostate-specific antigen; RP, radical prostatectomy; RT, radiotherapy.

We retrospectively reviewed the medical records of 2,290 patients with prostate cancer who underwent RP between 2004 and 2018 in a single institution. Among these patients, 138 (6%) had pathologically confirmed lymph node metastasis. Of those LNP patients, there were 8 patients with persistent elevated PSA and 86 patients with confirmed BCR (n = 86) after surgery. The exclusion criteria were as follows: lost to follow-up after treatment (n = 7); salvage treatment at another hospital (n = 5); maintained PSA at an undetectable level after surgery (n = 28); and observation after confirmed BCF (n = 4). Ultimately, 94 patients who received salvage treatment were included in this study (Fig 1). The study protocol was approved by the Institutional Review Board of Seoul National University Bundang Hospital (IRB No. B-2004/606-108).

### Treatments and follow-up

All patients underwent RP and PLND as initial treatments. Seventy-five patients (79.8%) received robotic-assisted laparoscopic prostatectomy and 19 patients (20.2%) were treated with retropubic RP. Of the 94 patients included in this study, 30 (31.9%) were identified as clinically node positive before surgery and 64 (68.1%) were negative. Most patients underwent limited PLND (n = 85; 90.4%), while the remainder (n = 9; 9.6%) were treated with extended PLND.

ADT consisted of luteinizing hormone releasing hormone (LHRH) agonist alone (n = 14; 37.8%) or LHRH agonist plus antiandrogen (n = 23; 62.2%). The median duration of ADT was 17.6 months [interquartile range (IQR), 11–26 months]. A combination of salvage ADT and RT was administered in 57 patients (60.6%). In this group, ADT was completed before the beginning of RT, and the median time interval between the two treatments was 1.8 months (IQR, 0–4 months). RT was administered with 6–15 MV photons from a linear accelerator using either 3-dimensional (3D) conformal RT (n = 10) or intensity-modulated RT (n = 47). External beam RT was delivered to whole pelvis followed by prostatic ± seminal vesicle bed boost. The median RT dose of 67 Gy (IQR, 66–70 Gy) was delivered to the clinical target volume. All patients were treated with conventional fractionation (1.8–2.0 Gy/fraction). The median duration of ADT in patients receiving combined therapy with RT was 20.4 months (IQR, 11–27 months).

After salvage treatment, patients underwent regular PSA measurements and diagnostic imaging studies (computed tomography of the abdomen and pelvis and/or magnetic resonance imaging of the pelvis, and bone scans) according to the physicians’ discretion.

### Statistical analysis

The duration of follow-up and timing of treatment failure were calculated based on the start date of salvage treatment. BCF was defined as two consecutive measured PSA values of greater than 0.2 ng/ml after surgery. We defined PSA recurrence after salvage treatment using the same criteria as that used for the initial BCF diagnosis, which referred to as ‘2^nd^ BCF’. CP was defined as radiographically confirmed disease (local recurrence or distant metastasis) during follow-up. Including both ‘2^nd^ BCF’ and ‘CP’ was defined as ‘Failure after salvage treatment’. Progression-free survival (PFS) was calculated as the time from salvage treatment initiation until 2nd BCF, CP, or death, whichever occurred first.

The chi-square test was used for comparative analysis between the two treatment groups: ADT alone vs. ADT with RT. The Kaplan-Meier method was used to evaluate the rates of 2^nd^ BCF, CP, and PFS, and P-values were calculated using the log-rank test. The Cox proportional hazard model was used for univariate and multivariate analyses to analyze factors affecting clinical outcomes and prognosis. All statistical analyses were performed using STATA software (version 14.0; Stata Corp, College Station, TX) with a significance level of < 0.05.

## Results

The median follow-up duration was 55 months (IQR, 35–97 months). We analyzed patients according to salvage treatment method, ADT alone versus ADT with RT. Most of the salvage treatments (n = 86; 91.5%) in this study were conducted within 1 year after surgery. The treatment strategy for each patient was implemented on the clinical judgment of the physicians.

Thirty-seven patients (39.4%) were treated with salvage ADT alone and 57 patients (60.6%) received ADT with RT. There were some differences in clinicopathological characteristics between the two treatment cohorts by inherent heterogeneous nature of retrospective study. The ADT alone group showed a statistically higher proportion of pre-salvage PSA ≥ 1.0 ng/ml and borderline significance of the number of positive lymph nodes ≥ 2 than the ADT with RT group. The detailed characteristics of the salvage treatment groups are shown in Table 1.

**Table 1:** Clinicopathological characteristics of the salvage treatment groups.

	ADT alone (N = 37)	ADT with RT (N = 57)			
Characteristics	No. (range)	%	No. (range)	%	*P*-value
Age, years (median)	68 (48–82)		63 (51–77)		0.120
Initial PSA (ng/ml)					0.203
< 20	12	32.4	26	45.6	
≥ 20	25	67.6	31	54.4	
Gleason score					0.130
< 8	16	43.2	16	28.1	
≥ 8	21	56.8	41	71.9	
Pathological tumor stage					0.160
T1-3a	11	29.7	13	22.8	
T3b	26	70.3	39	68.4	
T4	0	0	5	8.8	
Surgical margin status					0.590
Negative	13	35.1	17	29.8	
Positive	24	64.9	40	70.2	
Number of positive					0.076
lymph nodes					
< 2	23	62.2	45	78.9	
≥ 2	14	37.8	12	21.1	
Number of lymph nodes harvested	12 (3–29)		10 (1–29)		0.220
PSA nadir after surgery(ng/ml)					0.586
< 0.005	19	51.4	26	45.6	
≥ 0.005	18	48.6	31	54.4	
PSA at BCF (ng/ml)					0.346
< 0.5	12	32.4	24	42.1	
≥ 0.5	25	67.6	33	57.9	
Pre-salvage PSA (ng/ml)					0.029
< 1.0	11	29.7	30	52.6	
≥ 1.0	26	70.3	27	47.4	
Interval between BCF and salvage treatment (months)					0.314
< 2	24	64.9	31	54.4	
≥ 2	13	35.1	26	45.6	
Duration of ADT (months)					0.126
< 6	2	5.4	9	15.8	
≥ 6	35	94.6	48	84.2	

Abbreviations: ADT, androgen deprivation therapy; BCF, biochemical failure; PSA, prostate-specific antigen; RT, radiotherapy.

**Fig 2:**
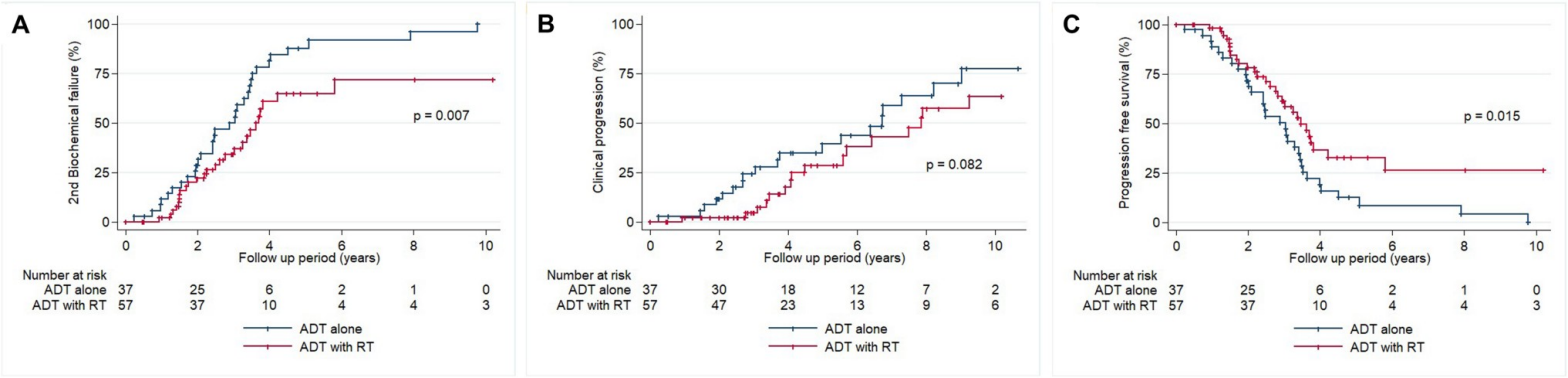
Actuarial treatment outcome curves of patients who received salvage ADT alone vs. ADT with RT. (A) Actuarial curves for 2^nd^ biochemical failure in the two groups according to salvage treatment method. (B) Actuarial curves for clinical progression in the two groups according to salvage treatment method. (C) Actuarial curves for progression-free survival according to salvage treatment method. Abbreviations: ADT, androgen deprivation therapy; RT, radiotherapy.

Table 2 shows the patterns of salvage failure in each treatment group. Among all 94 patients, 59 (62.8%) were identified for 2^nd^ BCF and 36 (38.3%) for CP during the follow-up period, respectively. The cumulative incidence of failure after salvage treatment in the ADT alone group was 33 (89.2%) of the 37 patients, and that of in the ADT with RT group was 26 (45.6%) of the 57 patients (HR, 22.4; 95% CI 5.43–92.1; P < 0.001). Among treatment failures, CP occurred more in patients who were treated with ADT alone than ADT with RT (54.1% and 28.1%, respectively), and bone metastasis was the most common type of distant metastasis in both groups. Fig 2A–2C show the actuarial rates of 2^nd^ BCF, CP and PFS after the salvage treatment. The ADT with RT group showed a significantly lower 2^nd^ BCF (Fig 2A, *P* = 0.007) and higher PFS (Fig 2C, *P* = 0.015) than the ADT alone group. CP did not significantly differ between the two treatment groups, but there was a better trend in the ADT with RT group (Fig 2B, *P* = 0.082).

**Table 2:** Patterns of treatment failure according to salvage treatment method.

	ADT alone (N = 37)		ADT with RT (N = 57)	
	No.	%	No.	%
Biochemical failure only	13	35.1	10	17.5
Clinical progression				
Local recurrence without distant metastasis	2	5.5	4	7.0
Distant metastasis	18	48.6	12	21.1
Bone	14		9	
Lung	1		0	
Liver	0		1	
Multiple organs	3		2	
Cumulative incidence of treatment failure	33	89.2	26	45.6

Abbreviations: ADT, androgen deprivation therapy; RT, radiotherapy.

**Fig 3:**
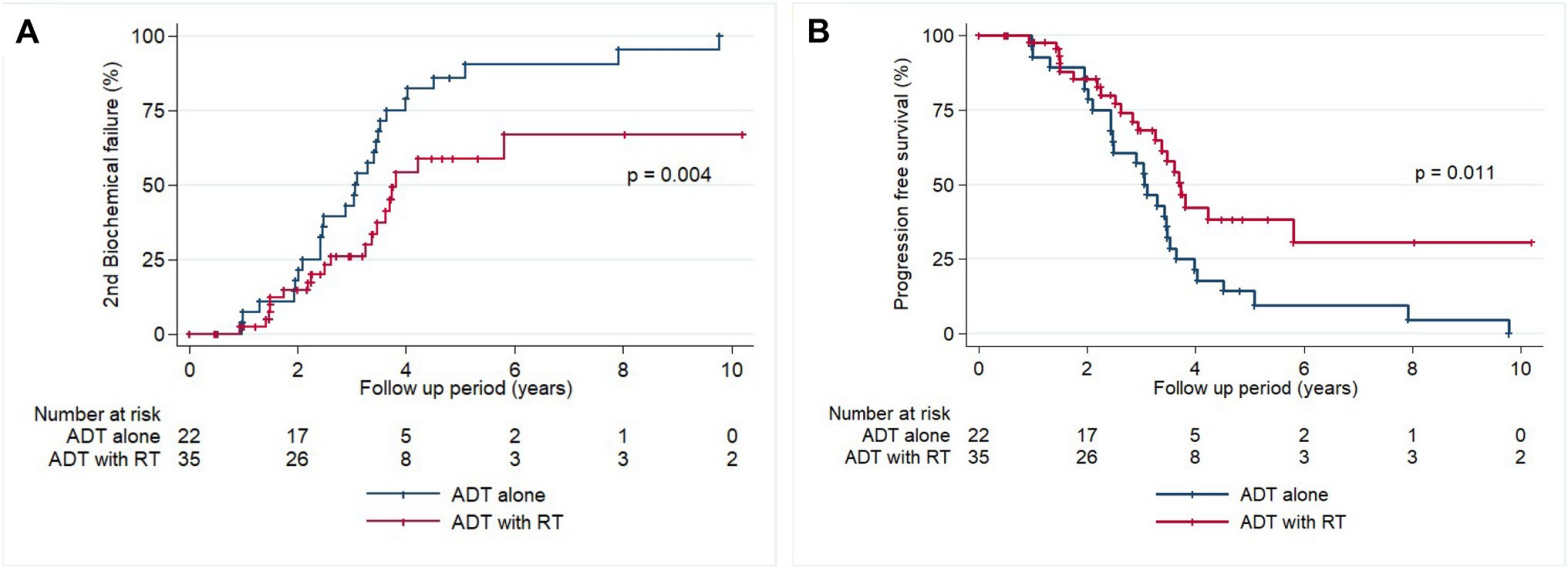
Actuarial curves for the subgroup patients with positive LN ≥ 2 or PSA nadir after RP ≥ 0.005 ng/ml. (A) Actuarial curves for 2^nd^ biochemical failure according to salvage treatment method. (B) Actuarial curves for progression-free survival according to salvage treatment method. Abbreviations: ADT, androgen deprivation therapy; LN, lymph node; PSA, prostate-specific antigen; RP, radical prostatectomy; RT, radiotherapy.

The results of the univariate and multivariate analyses of clinical and pathological factors are shown in Table 3. In the multivariate analyses, number of positive LN ≥ 2 and PSA nadir after RP ≥ 0.005 ng/ml were associated with poor 2^nd^ BCF, CP and PFS after salvage treatment. Patients with pathologically positive LN ≥ 2 showed a significant increase in 2^nd^ BCF (hazard ratio [HR], 2.00; 95% confidence interval [CI], 1.06–3.76; *P* = 0.031) and CP (HR, 2.28; 95% CI 1.01–5.11; *P* = 0.046) and decrease in PFS (HR, 1.92; 95% CI 1.03–3.58; *P* = 0.038). PSA nadir after RP also had a significant impact on the rates of 2^nd^ BCF (HR, 2.96; 95% CI 1.60–5.37; *P* = 0.001), CP (HR, 2.41; 95% CI 1.07–5.45; *P* = 0.034) and PFS (HR, 3.04; 95% CI 1.66–5.54; *P* < 0.001). ADT with RT treatment was significantly related to lower 2^nd^ BCF (HR, 0.52; 95% CI 0.30–0.89; *P* = 0.018) and higher PFS (HR, 0.54; 95% CI 0.32–0.94; *P* = 0.028) compared to ADT alone. In subgroup analyses, among patients with positive LN ≥ 2 or PSA nadir after RP ≥ 0.005 ng/ml, ADT with RT resulted in improvements in 2^nd^ BCF (Fig 3A; *P* = 0.004) and PFS (Fig 3B; *P* = 0.011) than ADT alone.

**Table 3:** Univariate and multivariate analysis of 2^nd^ biochemical failure, clinical progression, and progression-free survival after salvage treatment.

	2^nd^ Biochemical failure			Clinical progression			Progression-free survival		
	Univariate	Multivariate		Univariate	Multivariate		Univariate	Multivariate	
	*P*-value	*P*-value	HR (95% CI)	*P*-value	*P*-value	HR (95% CI)	*P*-value	*P*-value	HR(95% CI)
Initial PSA (ng/ml) ≥ 20 (vs. < 20)	0.062	0.203	1.53 (0.80–2.93)	0.143	0.776	1.13 (0.48–2.66)	0.072	0.222	1.49 (0.79–2.83)
Gleason score ≥ 8 (vs. < 8)	0.543	-	-	0.371	-	-	0.613	-	-
Pathological tumor stage ≥ T3b (vs. < T3b)	0.048	0.118	1.89 (0.85–4.20)	0.132	0.609	1.25 (0.53–2.96)	0.062	0.116	1.87 (0.86–4.09)
Number of positive LN ≥ 2 (vs. < 2)	0.003	**0.031**	2.00 (1.06–3.76)	0.010	**0.046**	2.28 (1.01–5.11)	0.004	**0.038**	1.92 (1.03–3.58)
Salvage Treatment: ADT with RT (vs. ADT alone)	0.008	**0.018**	0.52 (0.30–0.89)	0.111	0.291	0.68 (0.34–1.39)	0.017	**0.028**	0.54 (0.32–0.94)
PSA nadir after op (ng/ml) ≥ 0.005 (vs. < 0.005)	< 0.001	**0.001**	2.96 (1.60–5.48)	0.001	**0.034**	2.41 (1.07–5.45)	< 0.001	**< 0.001**	3.04 (1.66–5.54)
PSA at BCF (ng/ml) ≥ 0.5 (vs. < 0.5)	0.045	0.874	0.94 (0.43–2.06)	0.031	0.952	1.03 (0.38–2.78)	0.049	0.922	0.96 (0.45–2.07)
Pre-salvage PSA (ng/ml) ≥ 1.0 (vs. < 1.0)	0.010	0.136	1.92 (0.81–4.52)	0.012	0.205	1.89 (0.71–5.09)	0.010	0.116	1.97 (0.85–4.58)
Interval between BCF and salvage treatment (months) ≥ 2 (vs. < 2)	0.385	0.057	2.00 (0.98–4.06)	0.790	0.078	2.05 (0.92–4.57)	0.423	0.051	2.01 (0.99–4.04)
Duration of ADT (months) ≥ 6 (vs. < 6)	0.820	0.170	0.47 (0.16–1.39)	0.107	0.238	3.43 (0.44–26.5)	0.872	0.071	0.39 (0.14–1.08)

Abbreviations: ADT, androgen deprivation therapy; BCF, biochemical failure; LN, lymph node; PSA, prostate-specific antigen; RT, radiotherapy.

## Discussion

Several recent prospective randomized studies reported that there was no significant difference in treatment outcomes, including biochemical progression, between adjuvant and early salvage RT in high-risk prostate cancer [16–19]. These trials also showed that adjuvant RT increased the risk of genitourinary morbidity, suggesting that early salvage RT following RP seemed a preferable treatment for locally advanced prostate cancer. Additionally, according to the Japan Clinical Oncology Group 0410 results, salvage RT ± ADT group showed a significantly longer time to treatment failure than the salvage ADT alone group in localized prostate cancer patients with PSA failure after RP [15]. These studies support favoring salvage RT in prostate cancer patients with PSA failure. However, patients with LNP were excluded or only a few were included in these trials. In particular, there was no prospective study dealing with LNP prostate cancer patients to compare clinical outcomes of salvage treatment methods. Therefore, we evaluated the role of ADT ± salvage RT and prognostic factors in patients with LNP prostate cancer.

In the present study, 94 patients with LNP prostate cancer with BCR or persistent elevated PSA following RP received salvage treatment. The salvage therapy consisted of either ADT alone or ADT with RT. We found that ADT with RT was associated with better 2^nd^ BCF and PFS after salvage treatment. Also, patients with number of positive LN ≥ 2 or PSA nadir ≥ 0.005 ng/ml after RP showed significant improvements in clinical outcomes when treated with a combination of ADT with RT rather than ADT alone.

Several retrospective studies have reported that ADT with RT as an adjuvant treatment for LNP prostate cancer produced superior treatment outcomes. Da Pozzo et al. reported that adjuvant ADT with RT had a beneficial impact on BCF-free survival and cancer-specific survival (CSS) in node-positive prostate cancer (gain in the predictive model: 3.3% and 3%, respectively; all p < 0.001) [20]. In addition, Abdollah et al. reported that adjuvant ADT with RT in LNP prostate cancer was associated with improved cancer-specific mortality in patients with the following specific risk factors: (1) patients with ≤ 2 LNP, Gleason score ≥ 7, stage ≥ pT3b, or positive resection margin; or (2) patients with 3–4 LNP [21]. Similar to the results of adjuvant treatment analyzed in other studies, we confirmed that the combination of ADT and RT as a salvage treatment showed significant benefits in terms of 2^nd^ BCF and PFS.

According to the previously published data, adverse pathological features in prostate cancer include positive resection margin, seminal vesicle invasion, and extracapsular extension [22, 23]. In particular, Gleason score ≥ 8 and a high number of positive nodes in LNP prostate cancer are also known as aggressive pathologic characteristics [5]. However, most of the pathological factors that were examined in this study were not significantly related to the outcomes of salvage treatment for LNP prostate cancer. Instead, number of positive LN ≥ 2 was found adversely affecting 2^nd^ BCF, CP and PFS. Briganti et al. reported that 703 patients with node-positive prostate cancer with less than 2 LNs who underwent surgery showed significant benefits in terms of cancer-specific survival at 15 years (84% vs 62%; p <0.001) [24]. These results are consistent with the findings of our study and suggest that the number of metastatic LNs can be a significant risk factor for LNP prostate cancer.

Several studies have shown that pre-salvage PSA levels are significantly related to treatment outcomes [25–27]. Stephenson et al. reported that, when salvage RT was administered at PSA < 0.5 ng/ml, excellent biochemical control was obtained [26]. In addition, Stish et al. demonstrated that this cutoff value was associated with reduced incidence of distant metastasis, cancer-specific mortality, and mortality from all causes [27]. Furthermore, Bartkowiak et al. reported that salvage RT at PSA < 0.2 ng/ml was significantly correlated with accomplishing a post-salvage PSA nadir < 0.1 ng/ml and benefitted disease progression [25]. However, in our study, pre-salvage PSA was shown to be a significant factor in 2^nd^ BCF, CP, and PFS in univariate analysis, but not in multivariate analysis. Instead, we found that PSA nadir after RP could significantly affect clinical outcomes after salvage treatment. Shen et al. used an ultrasensitive PSA assay to evaluate the relapse rate of 906 prostate cancer patients who underwent RP [28]. Among them, 423 patients achieved PSA nadir less than 0.01 ng/ml, and those patients had a significantly lower risk of biochemical relapse. In addition, they recommended that patients who do not reach a sufficient PSA nadir may require early adjuvant or salvage intervention. In our study, patients in whom the PSA nadir after initial RP reached 0.005 ng/ml or less showed significantly better outcomes in terms of 2^nd^ BCF, CP, and PFS. Moreover, salvage ADT with RT was associated with reduced recurrence compared to ADT alone in patients with PSA nadir ≥ 0.005 ng/ml or with number of positive LN ≥ 2. These findings suggest that patients having such distinct characteristics after RP are at high risk of recurrence and that ADT with RT would be preferably recommended for salvage treatment in LNP prostate cancer patients.

This study had several limitations. First, it was a retrospective single-institutional study, which entailed an inherent bias related to the determination of the salvage treatment method (ADT or ADT with RT). Second, the entire cohort didn’t include enough patients to confirm the results. Third, most of the patients underwent limited PLND rather than extended PLND. Fourth, duration of ADT was not standardized and differed among patients. Last, the ADT alone group had a significantly higher proportion than pre-salvage PSA ≥ 1.0 ng/mL than the ADT with RT group. However, the choice of appropriate salvage treatment for each case was based on the physician’s judgment, since no clear consensus has yet been set on the treatment of LNP patients with BCR or persistent high PSA. Thus, a bias for selecting the treatment strategy was inevitable. Regarding the relatively small sample size, it was basically difficult to have a large number of BCR cases in LNP prostate cancer in a single institution. To overcome these shortcomings, we included all patients from 2004 to 2018 to cover as many patients as possible. On the other hand, there might be concerns that possible advance in treatment procedures or technologies may have affected the outcomes by spanning a fairly long time period. However, the clinical outcomes in this study would be reliable, because 1) the guidelines of treatment in LNP cohort have not changed significantly during that time period, 2) 82.5% of patients in the ADT+RT group underwent IMRT plan which could be considered as an advanced technology. Regarding the high proportion of limited PLND, it might have worries to affect negative influences on clinical results [29]. According to Bivalacqua et al., prostate cancer patients who received extended PLND showed significantly better 5-year BCF-free survival than those treated with limited PLND, as well as a trend towards improvement in the 10-year CSS rate [29]. However, the results on the therapeutic effect of extended PLND vary from study to study, and there is debate about its usefulness [30, 31]. We conducted multivariate analyses in the two groups (limited PLND vs. extended PLND) to figure out any difference affecting the outcomes in our cohorts, and there were no statistically significant results in 2nd BCF (HR = 0.95, P = 0.901), CP (HR = 0.94, P = 0.915), and PFS (HR = 0.92, P = 0.846). Also, it should be noted that the type of surgery may vary between physicians and even institutions. Next, the duration of salvage ADT in LNP prostate cancer could be differed among different studies and the optimal duration has not yet been proven in prospective trials [3, 15, 27]. Finally, differences in pre-salvage PSA level between the two treatment groups may have affected treatment outcomes. In particular, high pre-salvage PSA level in the ADT alone group could have led to worse treatment outcomes. These limitations need to be validated by strict guidelines in prospective studies and need to be verified. Despite these limitations, the results of this study are clinically meaningful due to inclusion of only LNP prostate cancer patients with BCR or persistence of elevated PSA. In addition, we evaluated pre-salvage treatment factors, such as a number of positive LN and PSA nadir value after initial RP, to predict clinical outcomes that would favor the combination of ADT with RT in the LNP patients following RP. Our novel findings may help physicians determine the optimal salvage treatment method for these high risk patients.

## Conclusions

In LNP prostate cancer patients with BCR or persistent high PSA, the combination of ADT with RT was associated with improved 2^nd^ BCF and PFS after salvage treatment. Patients with number of positive LN ≥ 2 or PSA nadir ≥ 0.005 ng/ml after RP may benefit more from the combination of ADT with RT as a salvage treatment.

## Supporting information

S1 File Dataset. (XLSX)
